# Concomitant Transarterial and Transvenous Embolization of a Pelvic Arteriovenous Malformation Using a New Liquid Embolic Agent, Squid-12 and Detachable Coils

**DOI:** 10.1155/2014/972870

**Published:** 2014-08-07

**Authors:** Aysun Erbahceci Salik, Filiz Islim, Ahmet Akgul, Barbaros Erhan Cil

**Affiliations:** ^1^Department of Radiology, Istanbul Bakirkoy Dr. Sadi Konuk Training and Research Hospital, 34147 Istanbul, Turkey; ^2^Department of Cardiovascular Surgery, Istanbul Bakirkoy Dr. Sadi Konuk Training and Research Hospital, 34147 Istanbul, Turkey

## Abstract

We describe a complex congenital pelvic AVM with multiple feeding arteries arising from the side branches of the right internal iliac artery and a single draining vein in a male patient. Concomitant transarterial and transvenous embolization with a new liquid embolic agent Squid-12 and metallic coils enabled a complete embolization at a single session. Squid-12 is composed of ethylene vinyl alcohol copolymers and its lower viscosity makes it a promising agent for the treatment of AVMs. The patient showed prompt resolution of the symptoms and complete devascularization of the AVM lesion was persisted on the 1-month control angiography. The patient was asymptomatic on the 6th month follow-up.

## 1. Introduction

Pelvic arteriovenous malformations (AVMs) are rare vascular lesions, which are characterized by multiple abnormal communications between the arterial and venous systems without an intervening capillary network [1–3]. In this region, these lesions are more frequently acquired secondary to neoplasms, pelvic trauma, and surgical procedures [1, 2, 4]. Congenital pelvic AVMs are uncommon, particularly in males with only a few cases which have been previously reported [2, 5]. Clinical presentation of this entity ranges from an asymptomatic large size vascular lesion on pelvic imaging studies to a life-threatening hemorrhage or congestive heart failure [6]. Scarcity, vague symptoms, and deep location of the lesions make this disease a diagnostic challenge.

Surgical treatment of pelvic AVMs either by ligation of the afferent arteries or by excision of the nidus has been reported to carry the risks of massive intraoperative hemorrhage, surrounding organ injury with high rates of recurrence and incomplete removal of the AVM nidus [6, 7]. Furthermore, ligation of the afferent arteries results in enlargement of the fistula, recruitment of the small feeding arteries, and makes the subsequent endovascular treatment more complicated or even impossible by blocking the transarterial access route. Therefore, endovascular treatment with various embolic and sclerosing materials has been shown to be an effective primary treatment option for management of patients with AVMs [6, 8]. The experience regarding the use of Onyx-18 which is composed of ethylene vinyl alcohol (EVOH) copolymers in the treatment of cerebral and peripheral vascular malformations has been growing within last decade [9, 10]. Squid (Emboflu, Gland, Switzerland) is a new non adhesive EVOH based liquid embolic agent which has been recently been made commercially available in Squid-12 and Squid-18 forms. Squid-12, due to its lower viscosity and possibly higher distal penetration capability, seems to have additional advantage in the embolization of AVMs. We report a male patient with a congenital pelvic AVM that was successfully treated at one session by concomitant transcatheter arterial and venous embolization using Squid-12 and metallic detachable coils.

## 2. Case Report

**Figure 1:**
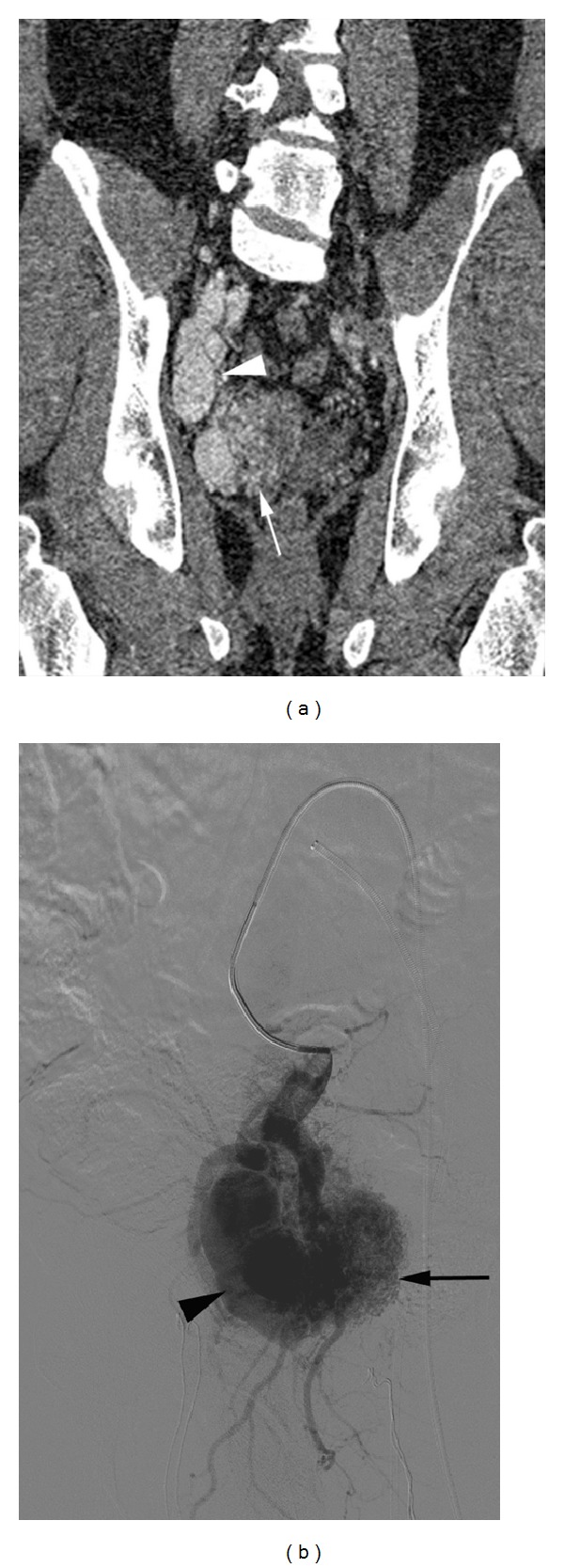
(a) Contrast-enhanced computed tomography in the arterial phase revealed a large pelvic arteriovenous malformation in the right lower quadrant. Multiple fine striations representing multiple feeding arteries (arrow) with early enhancement of a large venous pouch (arrowhead). (b) Selective arteriography of the right internal iliac artery anterior trunk revealed the nidus (arrow) with multiple fine feeders, dilation of the draining vein (arrowhead), and early drainage of the right internal iliac vein.

A 46-year-old man with no significant medical history presented with progressively increasing pelvic pain for 1 year and exacerbation for 1 month. Physical examination was normal. Laboratory data showed no abnormality. Ultrasonography of the pelvic region revealed a hypoechogenic mass in the right lower quadrant. Pelvic magnetic resonance imaging (MRI) revealed asymmetric multiple fine striations in the right pelvic cavity and dilation of the right internal iliac vein and artery. Computed tomography angiography (CTA) demonstrated a large pelvic AVM with multiple feeding arteries arising from the side branches of the right internal iliac artery especially from the anterior trunk which were draining into right internal iliac vein through a large venous pouch (Figure 1(a)). Because he had no history of pelvic trauma, neoplasm, or surgery we considered the AVM to be congenital anomaly. The patient was evaluated by a cardiovascular surgeon and interventional radiologist, and endovascular treatment was decided on because of high risk of intraoperative hemorrhage.

The procedure was performed after obtaining a written informed consent of the patient and under general anesthesia. Left common femoral artery and left common femoral vein were punctured simultaneously using the Seldinger technique and 6 F long introducer sheaths (Terumo Destination, Leuven, Belgium) were positioned. 5 F cobra catheters (Cordis Corporation, Bridgewater, NJ, USA) were inserted to access the right internal iliac artery and vein. A pelvic digital subtraction angiography (DSA) was performed. DSA confirmed the findings of the CTA and revealed a large pelvic AVM with multiple feeding arteries originating from the side branches of the right internal iliac artery especially from the anterior trunk. Multiple feeding arteries shunted a single large venous pouch and drainage occurred via the right internal iliac vein (Figure 1(b)).

**Figure 2:**
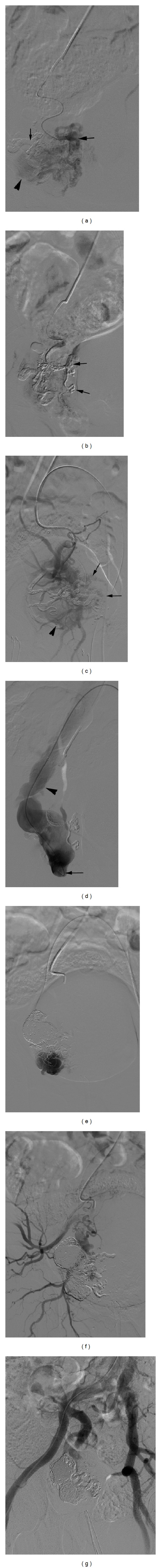
(a) Superselective arteriography of one of the feeding arteries (arrow) before Squid injection, venous pouch (arrowhead), and Squid cast adjacent to the catheterized feeding artery (thin arrow) were seen. (b) Superselective arteriography after Squid injection revealed Squid cast in the feeding artery (arrow). (c) Squid cast is visible on the selective arteriography of the right internal iliac artery anterior trunk after arterial Squid-12 injection. Venous pouch (arrowhead) was still visible because of fine arterial feeders (arrow). (d) A 2.7 F microcatheter was positioned in the venous pouch (arrow) and dilation of the draining vein (arrowhead) was seen on venography. (e) After deployment of 17 detachable metallic coils into the venous pouch selective contrast injection showed outflow occlusion and residual space in the venous pouch. (f) Completion arteriography revealed complete embolization of the AVM nidus. (g) Follow-up arteriography revealed complete devascularization of the AVM.

Feeding arteries, suitable to microcatheter insertion were superselectively catheterized using a 2.7 F microcatheter (Progreat, Terumo Medical, Leuven, Belgium). These feeding branches were embolized by injection of a total of 10.5 mL Squid-12 (Figures 2(a) and 2(b)). Injection was performed under fluoroscopic guidance and proximal reflux was considered as limitation. The control arteriography after arterial embolization revealed slower flow of the AVM but the venous pouch was still visible because of multiple fine arterial feeders which were inaccessible with microcatheter (Figure 2(c)).

Next, a 2.7 F microcatheter was positioned in the venous pouch through the cobra catheter placed via the venous access. Contrast injection confirmed the position of the microcatheter in the venous pouch of the AVM (Figure 2(d)). Subsequently, eight 0.020-inch detachable metallic coils (Penumbra Inc., Alameda, CA, US) and nine 0.018-inch fibered detachable metallic coils (Concerto, ev3 Endovascular, Plymouth, MN, USA) were deployed into the venous pouch leading to occlusion of the outflow drainage (Figure 2(e)). Residual space in the venous pouch adjacent to the AVM nidus was filled with Squid-12 until complete stasis was achieved. Coils obliterating the outflow of the venous pouch avoided the risk of leakage of the liquid embolic agent into the iliac vein and enabled the retrograde penetration of the Squid-12 into the fine arterial feeders. Completion right internal iliac arteriography confirmed the complete embolization of the AVM nidus (Figure 2(f)).

A follow-up DSA was performed 1 month after the procedure and complete devascularization and thrombosis of the AVM lesion were confirmed (Figure 2(g)). The clinical symptoms of the patient were completely resolved. There were no complications related to embolization procedure. On 6th month follow-up he was asymptomatic.

## 3. Discussion

Congenital AVMs of the pelvis are rare and challenging lesions. The natural history of pelvic AVMs is variable; they can remain asymptomatic for years and once they become symptomatic they may demonstrate rapid enlargement [2, 6]. Presenting symptoms include pelvic discomfort and pain, rectal pain and tenesmus, vaginal bleeding, haematuria, hemospermia, impotence, and orchitis [2, 5, 6]. High-output heart failure may occur due to malformations with large arteriovenous shunts. Observation of asymptomatic or mildly symptomatic lesions without any intervention has been suggested by several authors [4, 8]. Conversely, symptomatic and enlarging lesions should be treated promptly, since they may lead to life-threatening conditions such as massive bleeding and high-output heart failure. Furthermore, rapid enlargement of the lesions results in a more complicated treatment procedure.

Surgical treatment of AVMs has been reported to be unsuccessful as surgical eradication of the nidus of an AVM is rarely possible. Surgical treatment options include ligation of the feeding arteries and excision of the lesion. Many authors agree that simple ligation of afferent arteries has no value, since new collaterals develop rapidly to bypass the ligatured vessels [11]. Surgical resection of the affected area has been complicated by massive hemorrhage, adjacent organ damage, and recurrence [7]. In addition, ligation of the main feeding arteries blocks the catheter access and so usually prevents a subsequent endovascular treatment [6].

Because of the rarity of the pathology there is not a consensus on a standardized endovascular treatment strategy of these lesions. Embolization of the feeding arteries for the treatment of pelvic AVM lesions has been reported as case reports and series; relatively high clinical and radiological recurrence have been observed after transarterial embolization alone using various embolic materials [5, 6, 10, 11]. Jacobowitz et al. in their series, which is the largest study regarding pelvic AVMs [6], have reported that multiple embolization procedures (a mean of 2.4 procedures) were required for the treatment of patients with vascular malformations of the pelvis. We believe that combination of arterial and venous embolization is an efficacious combination for the treatment of patients with pelvic AVMs. Concomitant embolization of the feeding arteries and draining vein renders complete devascularization in a single session possible.

It is a topic of debate that transarterial or transvenous embolization should be performed first. We did not prefer transvenous embolization as a first procedure because, first of all, stagnated arterial flow after transvenous occlusion impairs nidal penetration of liquid embolic agent. Secondly, transvenous embolization has a theoretical risk of increased pressure in the nidus during the time which passed till the arterial embolization has been completed which may provoke nidus rupture especially for large AVM lesions with high volume flow.

Various embolic materials such as metallic coils, polyvinyl alcohol (PVA) foam particles, rapidly polymerizing acrylic adhesives, absolute ethanol, and ethylene vinyl alcohol copolymers have been used for the treatment of AVM lesions [6, 10, 12]. Metallic coils can be used for the embolization of the draining vein as we used in our case [12]. However, coils are ineffective for arterial embolization since they usually cannot reach nidus and can only provide proximal obstruction. In this case, coils were used to occlude the outflow of the venous pouch adjacent to the iliac vein, which permits safe injection of the Squid into the residual space of the venous pouch without risk of Squid migration into the iliac vein.

Liquid embolic agents have been reported to be useful for AVM treatment [6, 12, 13]. According to Jacobowitz et al., N-butyl cyanoacrylate is the most useful agent that forms a cast of the multiple small vessels near the nidus of the malformation [6]. However, when acrylic adhesives are used, it is difficult to control the level of occlusion. Embolization with an inappropriate mixture may result in proximal feeder occlusion or nontarget embolization to the pulmonary arteries. Embolization with PVA particles may induce rapid devascularization of the AVM lesions however, due to recanalization of the lesion in the long term, particle embolization usually results in failure.

Safety and efficacy of EVOH for the treatment of extracranial AVMs have previously been described in literature [9, 10]. It has been reported that Onyx-18 penetrates the nidal compartment effectively and forms an embolic cast adjacent to the nidus [9]. We used a new liquid embolic agent, Squid-12, for recent case which is composed of EVOH copolymers dissolved in dimethyl sulfoxide (DMSO) and micronized tantalum powder for radiopacity. It has 4 commercially available formulas with variable density and viscosity. Due to lower viscosity of Squid-12 than Squid-18 and Onyx-18, easier and more profound penetration into the microvessels of nidal compartment of AVM has been expected. The penetration of the Squid-12 was effective and consequently we could achieve a complete devascularization. Unlike other liquid embolic agents, EVOH copolymers do not adhere to the endothelial wall and catheter tip. However, reflux of the Onyx over the microcatheter has been a technical problem [9]. It may cause entrapment of the microcatheter within the reflux. We did not experience microcatheter entrapment during recent case. Additionally, EVOH copolymer injection is painful and necessitates general anesthesia.

We performed a follow-up DSA one month after the procedure. CTA has been used for follow-up after treatment of such lesions by many authors. CTA has the advantage of being a noninvasive modality; however CT artefacts of metallic coils and Squid may prevent visualizing minimal residual AVM vascularization. Therefore, a follow-up DSA was decided to be sure of complete devascularization of the AVM. Furthermore, high radiation exposure is another disadvantage of the CTA. All AVM lesions should be followed up for long periods and we planned annual clinical and iliac Doppler follow-ups to avoid radiation exposure.

In conclusion, pelvic AVM is a rare form of congenital vascular malformations with serious complications and variable hemodynamics. We performed transarterial and transvenous embolization concomitantly using Squid-12 and detachable metallic coils for embolization of a pelvic AVM and achieved complete cure with a single session. This technique may be an effective option when the AVM lesion consists of a single draining vein and multiple feeding arteries, and it may minimize the need for subsequent procedures. The ease of use and good penetration capability of this new liquid embolic agent make it a promising agent and it merits further clinical study.
